# The impact of viremia on organ failure, biomarkers and mortality in a Swedish cohort of critically ill COVID-19 patients

**DOI:** 10.1038/s41598-021-86500-y

**Published:** 2021-03-30

**Authors:** Josef D. Järhult, Michael Hultström, Anders Bergqvist, Robert Frithiof, Miklos Lipcsey

**Affiliations:** 1 Department of Medical Sciences, Zoonosis Science Center, Uppsala University, Uppsala, Sweden; 2 Department of Surgical Sciences, Anesthesia and Intensive Care Medicine, Uppsala University, Uppsala, Sweden; 3 Integrative Physiology, Department of Medical Cell Biology, Uppsala University, Uppsala, Sweden; 4 Clinical Microbiology, Department of Medical Sciences, Uppsala University, Uppsala, Sweden; 5 Clinical Microbiology and Hospital Infection Control, Uppsala University Hospital, Uppsala, Sweden; 6 Hedenstierna Laboratory, CIRRUS, Department of Surgical Sciences, Anesthesiology and Intensive Care, Uppsala University, Uppsala, Sweden

**Keywords:** SARS-CoV-2, Innate immunity, Acute inflammation, Viral infection

## Abstract

The spread of virus via the blood stream has been suggested to contribute to extra-pulmonary organ failure in Coronavirus disease 2019 (COVID-19). We assessed SARS-CoV-2 RNAemia (RNAemia) and the association between RNAemia and inflammation, organ failure and mortality in critically ill COVID-19 patients. We included all patients with PCR verified COVID-19 and consent admitted to ICU. SARS-CoV-2 RNA copies above 1000/ml measured by PCR in plasma was defined as RNAemia and used as surrogate for viremia. In this cohort of 92 patients 59 (64%) were invasively ventilated. RNAemia was found in 31 patients (34%). Hypertension and corticosteroid treatment was more common in patients with RNAemia. Extra-pulmonary organ failure biomarkers and the extent of organ failure were similar in patients with and without RNAemia, but the former group had more renal replacement therapy and higher mortality (26 vs 16%; 35 vs 16%, respectively, p = 0.04). RNAemia was not an independent predictor of death at 30 days after adjustment for age. SARS-CoV2 RNA copies in plasma is a common finding in ICU patients with COVID-19. Although viremia was not associated with extra pulmonary organ failure it was more common in patients who did not survive to 30 days after ICU admission.

Trial registration: ClinicalTrials NCT04316884.

## Introduction

The severe acute respiratory syndrome coronavirus 2 (SARS-CoV-2) causing Coronavirus disease 2019 (COVID‑19) is a pathogen primarily infecting the respiratory system. Although many cases are mild, some patients develop more severe disease ranging from respiratory failure via acute respiratory distress syndrome (ADRS) to multiple organ failure^1^.

Organs outside the respiratory system could be involved in the disease process through several potential mechanisms such as micro and macro-thromboembolism, hyper-inflammation, and complement activation^2^. Another plausible patho-physiological mechanism would be the spread of SARS-CoV-2 through the blood stream, i.e. viremia^3^. Although several case-reports suggest that viremia is present in some COVID-19 patients^4–6^, systematic data on occurrence of viremia in severe COVID-19 is scarce. Initial studies have reported that viremia was only seen in a minority of hospitalized COVID-19 patients^7,8^. Two later studies on 10 patients each reported viremia in 7 patients^9^ and 10 patients respectively^10^. In a recent study on 58 patients with mild to severe COVID-19 with highly sensitive polymerase chain reaction (PCR) quantification of plasma SARS-CoV-2, viremia was found in 74% of the patients^11^. The association between viremia and physiologic derangement or organ failure has not been reported.

We hypothesized that viremia is common and mediates organ failure in patients with severe COVID-19.

Given the pathophysiologic, therapeutic and disease control implications of viremia in COVID-19 patients, we conducted a study in cohort of patients with PCR verified COVID-19 admitted to ICU. SARS-CoV-2 RNAemia (hereafter RNAemia) in plasma was used as surrogate for viremia.

The primary aim was to assess the incidence and extent of RNAemia in this cohort of severe COVID-19 cases. We also wanted to assess the possible association between RNAemia and inflammation, organ failure and mortality in these patients.

## Materials and methods

This prospective observational study was approved by the National Ethical Review Agency (EPM; No. 2020–01,623). Informed consent was obtained from the patient, or relative if unable. The Declaration of Helsinki and its subsequent revisions were followed. The protocol of the study was registered a priori (ClinicalTrials ID: NCT04316884). STROBE guidelines were followed for reporting.

### Data collection and patient cohort

The study was performed at the General intensive care unit (ICU) at Uppsala University Hospital, a tertiary care hospital in Sweden, at the time of the study only admitting COVID-19 patients. All adult patients with COVID-19 admitted to the ICU, during March to June 2020, were screened for eligibility and asked for consent. COVID-19 was diagnosed with positive polymerase chain reaction (PCR) for SARS-Cov-2 on nasopharyngeal swabs^12,13^.

Apart from demographical data, clinical data were recorded prospectively including medical history, medications, physiological data, level of organ support and date of death. Simplified acute physiology score 3 (SAPS3)^14^, Sequential Organ Failure Assessment (SOFA) score^15^, and organ support data were collected as reported in the results. Blood samples were collected on admission to the ICU and daily during the ICU stay. Full blood count (FBC), plasma C-reactive protein (CRP), procalcitonin, IL-6, fibrin d-dimer, troponin and pro-brain natriuretic peptide-NT (pro-BNP-NT); kidney function tests: plasma creatinine and cystatin C; liver function tests: plasma bilirubin, alanine aminotransferase, aspartate aminotransferase (AST), alkaline phosphatase (ALP) were performed in the hospital central laboratory. FBC was analyzed on a Sysmex XN instrument (Sysmex, Kobe, Japan) while plasma CRP, ferritin, troponin I, kidney and liver markers were analyzed on an Architect ci16200 (Abbott Laboratories, Abbott Park, IL, US). Acute kidney injury (AKI) was defined according to the KDIGO AKI definitions^16^. IL-6 was measured by a commercial sandwich ELISA kit, (D6050, R&D Systems, Minneapolis, MN).

### Sample collection and virus detection

Peripheral blood was collected from patients with COVID-19 into EDTA-containing tubes and plasma was separated using centrifugation at 3000 g for 10 min. After separation, all plasma samples were stored at -80 °C. Total nucleic acid was extracted from 200 μl plasma samples using eMAG (Biomerieux) according to manufacturer’s instruction with an elution volume of 60 μl and stored at 4 °C.

Viral RNA in plasma was determined by real-time RT-PCR recognizing the SARS-CoV-2 N-gene with the 2019-nCoV N1 reagent set from the previously described protocol from Center for Disease Control (CDC) of the United States^13^. For reverse transcription and real-time PCR we used the Taqman Fast Virus 1-step Master Mix (ThermoFischer Scientific) according to the manufacturer’s instructions. The reactions were performed with a sample volume of 10 µl in a total volume of 25 µl. Primer and probe concentrations were as follows: Forward primer, 400 nM; Reverse primer, 800 nM; and Probe, 200 nM. The probe was labeled with Yakima Yellow as flurophore with internal ZEN and terminal 3IABkFQ as quenchers. The real-time PCR analysis was performed on a RotorGene Q instrument (Qiagen) with the software v2.3.1. The thermal cycling steps were: 50 °C for 15 min, 95 °C for 2 min, and 45 cycles of 95 °C for 15 s and 60 °C for 30 s.

For qualitative analysis, a Ct value of < 32 was defined as a positive result, and a Ct value of ≥ 32 was defined as a negative result. It should be noted that the threshold value is platform-dependent and as comparison, we use a four Ct steps higher value for the same reaction on QuantStudio 6 Pro (Applied Biosystems).

For quantitative analysis, we used imported standard curves based on previously determined PCR efficiency and adjustment against a reference point analysed in the same run. As external calibrator, we used the ISO 13,485 certified molecular standard Quantitative Synthetic SARS-CoV-2 RNA: ORF, E, N (VR-3276SD, ATCC,). The reaction showed linearity over 6 orders of magnitudes with 10^9^ copies/ml and 300 copies/ml as the upper and lower limits of quantitative detection, respectively. Since the quantification has limited precision close to the detection limit, we defined clinically significant RNAemia as > 1000 RNA copies/mL plasma.

### Statistics

The number of patients was defined by including all patients consenting to the study. Assuming that at least 20% of the patients would have RNAemia, we would have analyze samples from at least 78 patients to find a 10% difference with a power of 0.8.

Data are presented as median (IQR) or as number of observations (percent of total number of observations). To compare groups Mann–Whitney U test was used. Spearman Rank correlation test was used for assessing the association between continuous variables. Logistic regression was performed with organ failure, organ support and 30-day mortality as dependent variables with RNA copies and age as sole predictors to calculate odds ratios. As the number of observations were limited, we used age as a surrogate for the pre COVID-19 risk of death. Therefore, we also assessed if the RNA copies after adjusting for age was an independent predictor of these outcomes. We performed a sensitivity analysis defining RNAemia as RNA copies above the Limit of detection (> 300 RNA copies/mL plasma) when relevant. The proportion of missing data was low, < 10%, and was therefore not imputed. For calculations and figures, STATISTICA software, version 13.5 (TIBCO Software Inc, Tulsa, OK) was used. p < 0.05 was considered significant where relevant.

### Ethics approval

This prospective observational study was approved by the National Ethical Review Agency (EPM; No. 2020–01,623).

### Consent to participate

Informed consent was obtained from the patient, or next of kin if the patient was unable give consent. Informed consent was obtained for publication of aggregated data. No individual is included, thus Consent for publication is not applicable.

## Results

The clinical characteristics of the 92 patients included in the study are presented in Table 1. In short, all patients were adult ICU patients with one or more comorbidities, admitted 10 (8–12) days after the initial symptoms of COVID-19. No patients were treated with corticosteroids for COVID-19. 59 patients (64%) were invasively ventilated for respiratory failure.

**Table 1:** Patient demographic characteristics and comorbidities in the whole cohort as well as sub-cohorts according to detected SARS-CoV-2 virus RNA in plasma. RNAemia was defined as SARS-CoV-2 RNA copies > 1000/ml plasma.

	All patients (n = 92)	Patients with RNAemia (n = 31)	Patients without RNAemia (n = 61)	p-value
Female, n (%)	21 (23)	4 (13)	17 (28)	n.s
Age, years	62 (52–71)	64 (55–71)	59 (51–70)	n.s
BMI (kg/m^2^)	29 (26–33)	29 (27–33)	28 (25–33)	n.s
SAPS3	53 (47–58)	53 (49–57)	53 (46–58)	n.s
COVID-19 day at sampling	14 (12–16)	12 (11–15)	15 (13–16)	n.s
ICU day at sampling	4 (3–4)	3 (2–4)	4 (3–5)	0.001
Active or ex-smoker, n (%)	23 (24)	11 (35)	12 (17)	n.s
Comorbidities, n (%)				
Pulmonary disease	24 (26)	11 (35)	13 (21)	n.s
Hypertension	48 (52)	22 (71)	26 (43)	0.01
Heart failure	4 (4)	2 (6)	2 (3)	n.s
Ischemic heart disease	11 (12)	6 (19)	5 (8)	n.s
Diabetes mellitus	25 (27)	10 (32)	15 (25)	n.s
Malignancy	4 (4)	2 (6)	2 (3)	n.s
Medications, n (%)				
Corticosteroid treatment prior to admission	11 (12)	8 (26)	3 (5)	0.035
RAAS inhibitor treatment prior to admission	36 (39)	16 (52)	20 (33)	n.s
Oral anticoagulant treatment prior to admission	17 (18)	9 (29)	8 (13)	n.s
Vital signs on ICU admission				
Respiratory rate (/min)	28 (23–36)	29 (23–36)	28 (25–35)	n.s
Heart rate (/min)	89 (77–100)	88 (78–97)	93 (77–102)	n.s
Mean arterial pressure (mmHg)	89 (81–99)	89 (77–98)	91 (84–102)	n.s
Body temperature (°C)	38.0 (37.5–38.7)	38.1 (37.5–38.5)	38.0 (37.6–38.7)	n.s

Data are expressed as n (%) or median (interquartile range, IQR). Groups compared with Z-test or Mann-Whiney U test. Abbreviations: BMI: Body mass index, ICU: intensive care unit, RAAS blockade: renin–angiotensin–aldosterone system inhibitors such as angiotensin converting enzyme inhibitor/angiotensin receptor blocker, SAPS3: Simplified acute physiology score 3.

RNAemia was found in 31 (34%) of the patients, but SARS-CoV-2 RNA in plasma was detected in 58 (63%) patients. To exclude the possibility of false positives due to unspecific reactions, the specificity was verified with 25 anonymized plasma samples from unrelated patients without covid-19 (data not shown).

Patients with RNAemia had hypertension in their previous medical history more commonly than patients without RNAemia. Also, more RNA copies were found in plasma in patients with hypertension vs. those without (2.78 (2.48–3.55) vs. 2.48 (2.48–2.95) Log copies/mL, p = 0.01) and those with vs. without premorbid corticosteroid treatment (3.43 (2.95–3.52) vs. 2.48 (2.48–3.28) Log copies/mL, p = 0.02).

**Figure 1:**
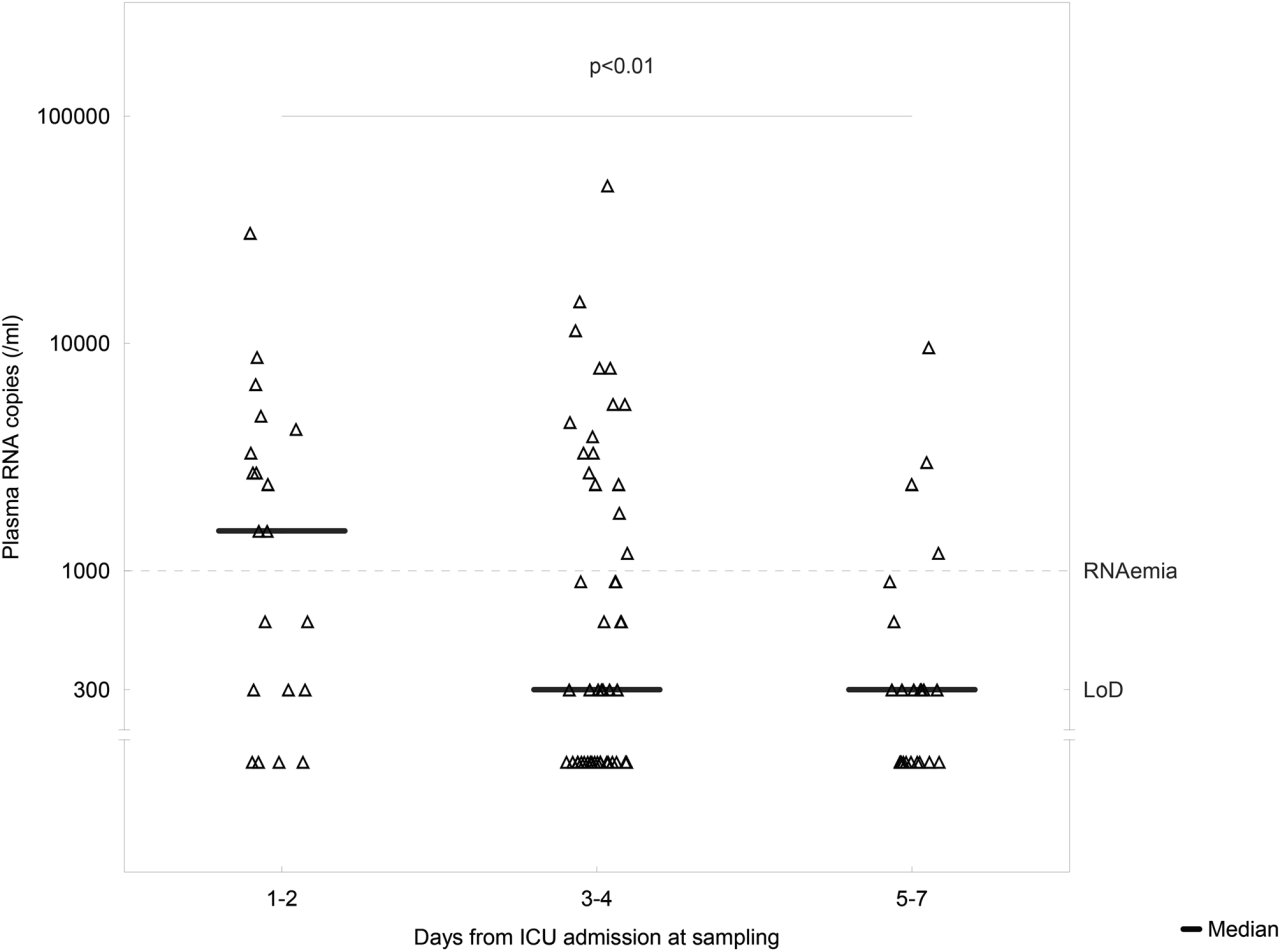
SARS-CoV-2 RNA levels in plasma on the ICU day of sampling. Mann–Whitney test was used to compare RNA levels on ICU day 1–2 and ICU day 5–7. The p-value denotes difference in RNA copies from ICU days 1–2 to 5–7.

In patients sampled ICU day 1–2, RNA copy levels in plasma were higher than those sampled ICU day 5–7 (p = 0.014, Fig. 1). The time from the first symptoms and time from ICU admission to sampling was inversely associated with plasma RNA copy levels (rho = -0.34, p = 0.001 and rho = -0.26, p = 0.01 respectively).

### Biochemistry

IL-6 levels were higher in the patients with vs. those without RNAemia (p = 0.04, Table 2) and IL-6 levels correlated weakly with RNA copies/mL plasma (rho = 0.23, p = 0.045). On the other hand, no differences were seen in patients with and without RNAemia in a broad palette of biomarkers representing full blood count, coagulation, inflammation, hepatic and cardiac injury and function, as well as renal function. Plasma RNA copy levels were not associated with any of these biomarkers as rho was < 0.2 in all these correlations (data not shown).

**Table 2:** Biochemistry data of patients on the day of sampling for detection of SARS-CoV-2 RNA in plasma. RNAemia was defined as SARS-CoV-2 RNA copies > 1000/ml plasma.

	Patients with RNAemia (n = 29)	Patients without RNAemia (n = 59)	p-value
Blood hemoglobin (g/L)	120 (109–130)	119 (103–126)	n.s
Blood leukocyte count (× 10^9^/L)	7.3 (6.0–9.6)	8.3 (6.3–11)	n.s
Blood neutrophil granulocyte count (× 10^9^/L)	5.9 (5.1–7.4)	5.8 (4.8–8.8)	n.s
Blood lymphocyte count (× 10^9^/L)	1.0 (0.65–1.3)	1 (0.7–1.3)	n.s
Blood platelet count (× 10^9^/L)	270 (199–334)	307 (227–380)	n.s
Plasma C-reactive protein (mg/L)	210 (131–309)	216 (141–299)	n.s
Plasma procalcitonin (µg/L)	0.6 (0.3–1.6)	0.53 (0.26–1.8)	n.s
Plasma interleukin-6 (ng/L)	180 (114–331)	108 (58–272)	0.046
Plasma ferritin (µg/L)	1539 (545–2571)	2105 (971–3052)	n.s
Plasma fibrin D-dimer (mg/L FEU)	1.6 (1.1–3.0)	1.7 (1.3–3.1)	n.s
Plasma ALT (µkat/L)	0.67 (0.42–0.99)	0.93 (0.45–1.48)	n.s
Plasma AST (µkat/L)	1.3 (0.71–1.79)	1.19 (0.73–1.61)	n.s
Plasma bilirubin (µmol/L)	10 (8–11)	10 (8–15)	n.s
Plasma lactate dehydrogenase (µkat/L)	6 (5–8)	6 (5–7)	n.s
Plasma N-terminal pro-BNP (ng/L)	360 (233–504)	340 (121–842)	n.s
Plasma Troponin I (ng/L)	11 (7.1–42)	8.2 (3.9–25)	n.s
Plasma creatinine (µmol/L)	86 (64–103)	71 (61–100)	n.s
eGFR_CystC_ (mL/min/1.73 m^2^ BSA)	58 (42–76)	58 (40–72)	n.s
eGFR_Crea_ (mL/min/1.73 m^2^ BSA)	67 (51–83)	75 (55–80)	n.s

Data are expressed as median (interquartile range, IQR). Groups compared with Mann-Whiney U test. Laboratory data missing for 4 cases. Abbreviations: ALT: alanine aminotransferase, AST: aspartate aminotransferase, ALP: alkaline phosphatase, BNP: Brain natriuretic peptide, BSA: Body surface area, eGFR_CystC_: Estimated glomerular filtration rate from plasma cystatin C, eGFR_Crea_: Estimated glomerular filtration rate from plasma creatinine, FEU: fibrinogen equivalent units, SOFA: Sequential Organ Failure Assessment.

### Outcome

There was no difference between patients with or without RNAemia in overall organ failure measured by maximum SOFA score, or the incidence of AKI, or the incidence of decreased level of consciousness or the incidence of critical illness weakness (Table 3). Renal replacement therapy (RRT) was more common (p = 0.04) and mortality at 30 days was higher (p = 0.04) in patients with RNAemia than patients without RNAemia. There was no difference in the proportion of patients receiving invasive ventilation or vasopressor treatment, and neither was there any difference in ventilator free days, vasopressor free days or RRT free days in patients with or without RNAemia. Patients that did not survive to 30 days had more RNA copies in plasma than those who did (3.38 (2.48–3.68) vs. 2.48 (2.48–3.18) Log copies/mL, p = 0.02).

**Table 3:** Patient organ failure, organ support and mortality during ICU stay according to detected SARS-CoV-2 virus RNA in plasma. RNAemia was defined as > 1000 SARS-CoV-2 RNA copies > 1000/ml plasma.

	All patients (n = 92)	Patients with RNAemia (n = 31)	Patients without RNAemia (n = 61)	p-value
Maximum SOFA score	9 (7–11)	9 (7–11)	9 (7–11)	n.s
AKI_crea_, n (%)	57 (62)	19 (61)	38 (62)	n.s
GCS < 14	18 (20)	6 (19)	12 (20)	n.s
Critical illness weakness, n (%)	10 (11)	6 (19)	4 (6)	n.s
Invasive ventilation, n (%)	59 (64)	20 (65)	39 (64)	n.s
Ventilator free days	25 (19–30)	24 (16–30)	26 (20–30)	n.s
Vasopressor treatment, n (%)	60 (65)	20 (65)	40 (66)	n.s
Vasopressor free days	26 (21–30)	25 (19–30)	26 (22–30)	n.s
Renal replacement therapy, n (%)	14 (15)	8 (26)	6 (10)	0.044
Renal replacement therapy free days	30 (30–30)	30 (28–30)	30 (30–30)	n.s
Mortality at 30 days, n (%)	21 (23)	11 (35)	10 (16)	0.039

Data are expressed as n (%) or median (interquartile range, IQR). Groups compared with Z-test or Mann-Whiney U test. AKI_crea_: Acute kidney injury defined by plasma creatinine, GCS: Glasgow coma scale.

**Figure 2:**
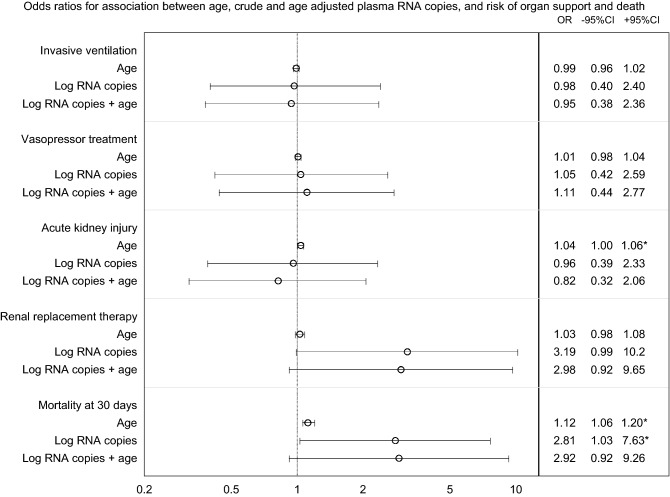
Odds ratios from logistic regression assessing the association between age, crude and age adjusted RNAemia in plasma, and risk of organ support and death. RNAemia was defined as SARS-CoV-2 RNA copies > 1000/ml plasma. * denotes p < 0.05 for odds ratio.

The presence of RNAemia in patients was not a predictor of organ support or AKI. Patients with RNAemia had increased crude risk of death. This effect was not present after adjustment for age (Fig. 2).

### Sensitivity analysis

Data in Tables 1–3 and Fig. 2 was also assessed with RNAemia defined as positive results, with cycle thresholds below the cutoff point, without changing the conclusions of the study (data not shown).

## Discussion

### Key findings

In this cohort of COVID-19 patients with respiratory failure requiring intensive care almost one third of the patients had RNAemia and almost two thirds had detectable SARS-CoV-2 RNA in their plasma. Although the level of RNA copies in plasma decreased during the first week of ICU care, RNAemia was still common up to seven days after ICU admission. Higher levels of RNA copies were found in patients with hypertension, and corticosteroid treatment. Despite substantial multiple organ failure, as suggested by SOFA score, the level of RNA copies in plasma was not associated with organ failure outside the respiratory system assessed by the patient’s biochemistry other than higher IL-6 levels in plasma. Although RRT was more common in patients with RNAemia this was not linked to AKI. The RNAemia did not predict level of organ support in this cohort. However, mortality was higher in patients with RNAemia.

### Relation to other studies

RNAemia in COVID-19 patients seen in this cohort and its relation to illness severity has been reported previously. In a small study on 10 severe COVID-19 patients it was reported that 7 of 10 had viremia^9^. Moreover some data indicate, similar to our findings, that viremia can be persistent and be present several weeks after the onset of illness^17^.

There was no association between illness severity and the level of RNAemia in our study. This is in line with another study with 41 patients, where although the ratio of patients with viremia was low, it was similar in non-critically and critically ill patients^8^. Yet, in the largest of the previous studies viremia was strongly associated with the admission to intensive or intermediate care^11,18^. Viremia per se may be a marker of severe disease as studies of hospitalized patients report lower ratios of viremic patients^7^ than studies with critically ill patients^10^. One possible explanation could be that in critically ill COVID-19 patients with respiratory failure, viremia does not lead to additional burden of illness in terms of distant organ failure. This is supported by our previously reported data from COVID-19 patients with high prevalence of AKI, that SARS-CoV-2 RNA in urine is a rare finding^19^.

In this study the level of RNA copies in plasma was higher in patients with hypertension and corticosteroids. This is in line with findings in larger cohorts^20,21^, but it raises the question whether RNAemia is a mediator of disease or merely a marker of disease severity.

The cohort in the current study consisted of ICU patients with severe respiratory failure supported by high-flow nasal prongs, non-invasive ventilation or invasive ventilation. Extra-pulmonary organ failure was present since SOFA score indicates multiple organ failure in the majority of the patients. Intuitively viremia could be proposed as a mechanism of distant organ damage. However, neither the presence, nor the extent of RNAemia was linked to organ failure outside the respiratory system. Although RRT was more common in patients with RNAemia a similar effect of RNAemia was not seen for AKI. In contrast to our findings a recent publication reports strong relationship between respiratory failure, cardiac injury, renal damage and RNAemia^22^. Yet as with studies reporting an association between RNAemia and the illness severity, one could speculate that when a certain level of illness is induced by the SARS-CoV-2 virus, derangement of biochemistry and the development of organ damage may be mediated by already triggered inflammatory systems rather than virus replication per se. However, this does not preclude that RNAemia is a marker of local virus load, especially in the lungs.

Our and other studies^22–24^ have shown that RNAemia was a predictor of death. In this cohort the effect of RNAemia was not present when adjusting for age, a surrogate for pre COVID-19 risk of death. As the death in COVID-19 is often due to respiratory failure^21,25^ and the burden of chronic diseases^20,26^, viremia mediated extra-pulmonary organ failure may not be the key mechanism of death.

### Strengths and limitations

As far as we know this is the first report of plasma SARS-CoV-2 RNA levels in relation to duration and severity of organ failure in a cohort of critically ill patients. We also included all patients admitted to the only ICU in the county unless they did not consent to the study, which was less than 10% of the patients, decreasing selection bias. An additional strength is the prospectively collected high resolution patient data covering all major organ systems. Finally these patients presented with the most severe forms of COVID-19 enabling us to study extra-pulmonary organ failure and its relation to RNA copy levels in plasma. Finally, we used a quality assured molecular standard that gives high reproducibility and comparability with other studies using the same method.

The study findings are limited by the method of virus detection. Although one would assume that presence of SARS-CoV-2 RNA in plasma indicates presence of SARS-CoV-2 virus, the PCR method only detects a fragment of the RNA, thus not differentiating between live virus and RNA fragments from non-infective virions. Ideally the presence of live virus would be confirmed by virus culturing, but that was not within the scope of this study. We also conclude that RNAemia was not linked to organ failure indicating other mechanisms than viral replication, but we cannot entirely exclude that some of the organ failure is triggered by direct viral spread through the blood stream. Finally, although the number of patients in this study is relatively large for ICU COVID-19 cohorts, the study is underpowered to exclude other than major effects in variables that were measured with high precision. For example, the possible association between RNAemia and renal replacement therapy will need to be investigated in larger cohorts of patients.

### Clinical implications

The most solid finding of this study is that in spite of the abundant RNAemia seen in this ICU cohort the effect of RNAemia was not associated to organ failure and the association to death was weak. Previous studies indicate that RNAemia in non-ICU patients can predict illness severity and outcome, but our results do not support the possibility of such a prediction among ICU patients. Moreover, our findings could indicate that blood of patients with severe COVID-19 potentially has considerable virus load and should be handled accordingly. Furthermore, RNAemia in this study may indicate the presence of live SARS-CoV-2 virus in patients up to a week after ICU presentation. This may be considered when starting therapies with potential immunosuppressive effect within the first week of presentation to health care. Both data from a case report^4^ and a large randomized trial^27^ suggest that the timing of immunosuppressive treatment in COVID-19 has major impact on outcome.

### Future research

There is limited knowledge on the extent and duration of viremia in COVID19 and are areas to explore. The patho-physiologic significance of RNAemia and whether it represents actual viremia as well as the pathophysiologic role of RNAemia in extra-pulmonary organ failure are important issues to investigate.

### Conclusions

RNAemia measured as SARS-CoV2 RNA copies in plasma is common in ICU patients with COVID-19 up to a week after ICU admission. Although RNAemia was not associated with extra pulmonary organ failure, patients with RNAemia had higher mortality at 30 days.

## Data Availability

Data is available upon reasonable request.
